# Molecular network-based identification of competing endogenous RNAs and mRNA signatures that predict survival in prostate cancer

**DOI:** 10.1186/s12967-018-1637-x

**Published:** 2018-10-04

**Authors:** Ning Xu, Yu-Peng Wu, Hu-Bin Yin, Xue-Yi Xue, Xin Gou

**Affiliations:** 1 Department of Urology, The First Affiliated Hospital of Chongqing Medical University, No. 1 Youyi Rd., Yuzhong District, Chongqing, 400016 China; 2 Departments of Urology, The First Affiliated Hospital of Fujian Medical University, Fuzhou, 350005 China

**Keywords:** Competing endogenous RNAs, Prostate cancer, 4-mRNA signature, Survival

## Abstract

**Background:**

The aim of the study is described the regulatory mechanisms and prognostic values of differentially expressed RNAs in prostate cancer and construct an mRNA signature that predicts survival.

**Methods:**

The RNA profiles of 499 prostate cancer tissues and 52 non-prostate cancer tissues from TCGA were analyzed. The differential expression of RNAs was examined using the edgeR package. Survival was analyzed by Kaplan–Meier method. microRNA (miRNA), messenger RNA (mRNA), and long non-coding RNA (lncRNA) networks from the miRcode database were constructed, based on the differentially expressed RNAs between non-prostate and prostate cancer tissues.

**Results:**

A total of 773 lncRNAs, 1417 mRNAs, and 58 miRNAs were differentially expressed between non-prostate and prostate cancer samples. The newly constructed ceRNA network comprised 63 prostate cancer-specific lncRNAs, 13 miRNAs, and 18 mRNAs. Three of 63 differentially expressed lncRNAs and 1 of 18 differentially expressed mRNAs were significantly associated with overall survival in prostate cancer (P value < 0.05). After the univariate and multivariate Cox regression analyses, 4 mRNAs (HOXB5, GPC2, PGA5, and AMBN) were screened and used to establish a predictive model for the overall survival of patients. Our ROC curve analysis revealed that the 4-mRNA signature performed well.

**Conclusion:**

These ceRNAs may play a critical role in the progression and metastasis of prostate cancer and are thus candidate therapeutic targets and potential prognostic biomarkers. A novel model that incorporated these candidates was established and might provide more powerful prognostic information in predicting survival in prostate cancer.

## Background

In men, prostate cancer remains the second leading cause of deaths due to cancer in the US [1]. Approximately 26,000 men were expected to die from prostate cancer in 2016 [2]. Siegel et al. [2] also estimated that many patients with advanced prostate cancer will develop castration-resistant prostate cancer (CRPC). Previous studies [3–6] have reported that there are several treatment options for CRPC, including chemotherapy, androgen receptor-targeted agents, and radiopharmaceuticals. Nevertheless, there are currently no effective biomarkers for the early diagnosis and treatment of prostate cancer.

Morphological, immunological, and molecular features have been used to predict the progression and prognosis of prostate cancers [7, 8]. Over the past several decades, urologists have devoted much effort toward identifying prostate cancer-related protein-coding genes [9]. However, only approximately 2% of all transcripts in mammals are protein-coding RNAs [10]. Thus, the functions of non-coding RNAs should be examined [11]. Previous studies [12–16] proposed a competing endogenous RNA (ceRNA) hypothesis, which described an intricate post-transcriptional regulatory network in which mRNAs, lncRNAs, and other RNAs act as natural miRNA sponges to weaken the function of miRNA via sharing one or more miRNA response elements.

In this study, a ceRNA network was constructed to identify the ceRNAs that are involved in prostate cancer using data from the TCGA database. The RNA profiles of 499 prostate cancer tissues and 52 non-prostate cancer tissues were analyzed. Finally, a prostate cancer-associated ceRNA network was established, based on our bioinformatics prediction and correlation analysis, consisting of 63 lncRNAs, 13 miRNAs, and 18 mRNAs. We examined the functions of the differentially expressed miRNAs that we identified and developed a novel model using several candidates to predict survival in prostate cancer patients. This study aimed to identify prostate cancer-specific RNAs as ceRNAs that regulate target genes and are involved in the pathogenesis and prognosis of prostate cancer.

## Methods

### Data collection

RNA profiles of prostate cancer and control samples were downloaded from the genomic data commons (GDC) data portal and the cancer genome atlas (TCGA, https://tcga-data.nci.nih.gov/tcga/) database. A total of 551 samples were collected, comprising 499 primary prostate cancer samples and 52 normal solid tissue samples.

### Differential gene expression analysis

mRNA, lncRNA, and miRNA expression in the prostate cancer samples were analyzed using the RNASeqV2 and Illumina HiSeq 2000 miRNA sequencing platforms. Samples were divided into prostate cancer tissues versus adjacent non-tumor tissues to identify differentially expressed RNAs using edgeR. Differences in the expression of each RNA between prostate cancer and adjacent non-tumor tissue were expressed as fold-change and the associated P value. Downregulated and upregulated RNAs were defined as those that decreased and increased by a fold-change of > 1.5, respectively, with an FDR-adjusted P of < 0.05.

### Construction of the ceRNA network

The regulatory network was constructed using data on the mRNAs, lncRNAs, and miRNAs. First, prostate cancer-specific RNAs, including mRNAs, lncRNAs, and miRNAs, were filtered. Downregulated and upregulated RNAs were assigned fold-changes > 1.5 with FDR-adjusted P < 0.05. Then, the mRNAs that were targeted by miRNAs were predicted using Targetscan (http://www.targetscan.org/), miRTarBase (http://mirtarbase.mbc.nctu.edu.tw/), and miRDB (http://www.mirdb.org/). Next, miRanda Tools (http://www.microrna.org/microrna/home.do) was used to predict the interactions between lncRNAs and miRNAs. Finally, miRNAs that regulated the expression of both lncRNAs and mRNAs were selected for construction of the ceRNA network using Cytoscape v.3.8.5.

### Survival analysis and definition of mRNA-related prognostic model

The association between differentially expressed mRNAs and overall survival was evaluated by univariate Cox proportional hazards regression analysis using the R survival package. Only mRNAs with P < 0.01 were considered to be candidates and selected for multivariate Cox regression analysis. The best explanatory and most informative predictive model was identified using Akaike Information Criterion (AIC), which assesses the goodness of fit of a statistical model.

### Gene ontology and pathway analysis

To understand the underlying biological processes and pathways between differentially expressed genes in the ceRNA network, the database for annotation, visualization, and integrated discovery (DAVID) (http://david.abcc.ncifcrf.gov/) was used to perform functional enrichment analysis. Then, significantly differentially expressed mRNAs were analyzed in the gene ontology (GO) database (http://www.geneontology.org). Finally, significantly enriched GO terms were selected to analyze their biological function. The kyoto encyclopedia of genes and genomes (KEGG; http://www.kegg.jp/) was used to perform the pathway enrichment analysis.

### Survival analysis of key members in the ceRNA network

The clinical data on the patients were combined with prostate cancer data in TCGA to evaluate the prognostic value of differential RNAs in the ceRNA network. Survival curves were generated using the survival package in R for samples with differentially expressed mRNAs, lncRNAs, and miRNAs. Survival was analyzed by Kaplan–Meier method, and P values < 0.05 were considered to be significant.

## Results

### Identification of significantly differentially expressed lncRNAs

**Fig. 1:**
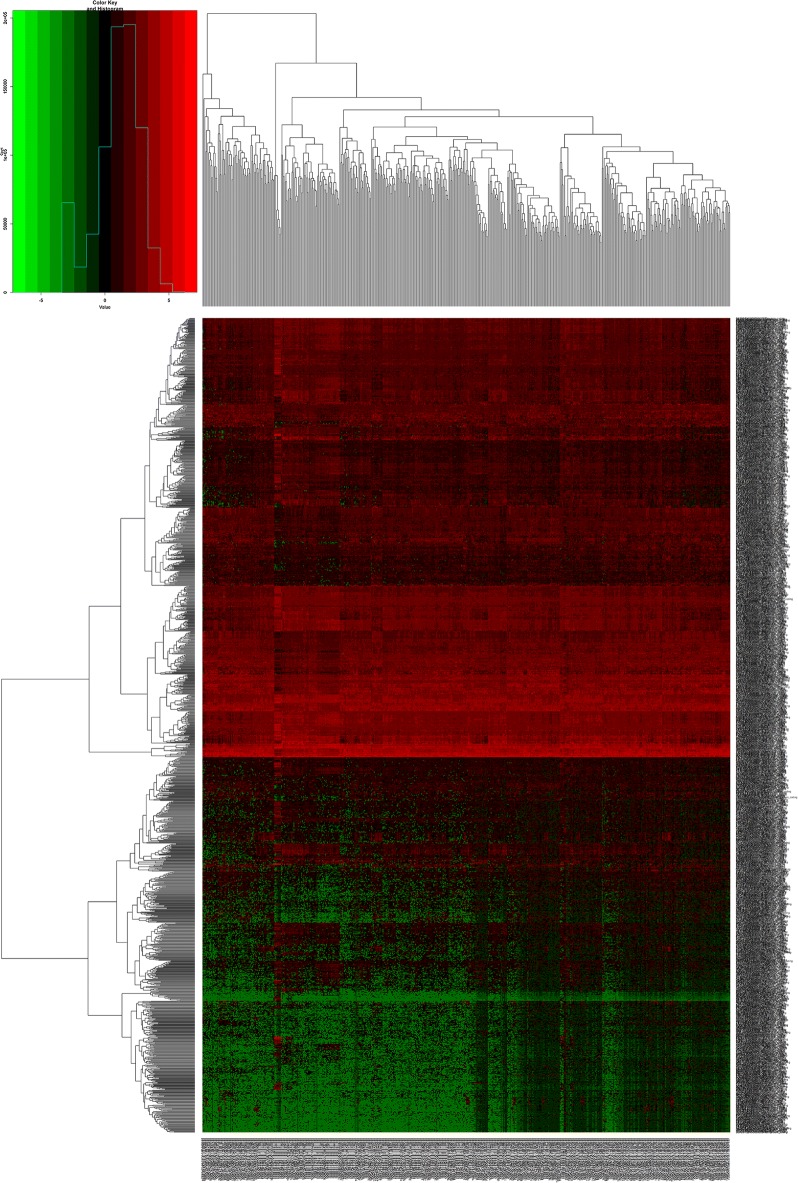
Heat maps of differentially expressed messenger RNAs (mRNAs) in prostate cancer

In this study, 551 samples were obtained from the TCGA database. Differential expression was analyzed by comparing the expression of 14,254 lncRNAs in prostate cancer and adjacent normal prostate tissues in the TCGA database. Fold-change > 1.5 and P value < 0.05 were set as cutoffs to identify significantly differentially expressed lncRNAs. As a result, 773 differentially expressed lncRNAs between prostate cancer and adjacent normal prostate tissue were obtained—of which 414 were upregulated and 359 were downregulated (Fig. 1; Table 1).

**Table 1:** Top differential mRNAs for prostate cancer

	logFC	logCPM	P value	FDR
SERPINA5	− 6.78954	4.392568	0	0
MFSD2A	− 5.97025	3.451485	0	0
ACSL6	− 4.99597	2.404013	1.15E−299	6.83E−296
MCF2	− 5.26855	0.926199	5.45E−264	2.43E−260
EMX2	− 6.79059	2.24242	3.15E−261	1.12E−257
HOXB8	− 6.24965	1.044248	8.97E−252	2.67E−248
CLDN2	− 7.91887	3.217525	1.31E−248	3.34E−245
AKR1B1	− 3.87736	5.710326	1.39E−239	3.11E−236
SPINK2	− 7.41992	2.675429	9.55E−238	1.89E−234
CYP19A1	− 5.40444	− 1.22883	8.73E−236	1.56E−232

### Identification of significantly differentially expressed mRNAs and miRNAs

**Fig. 2:**
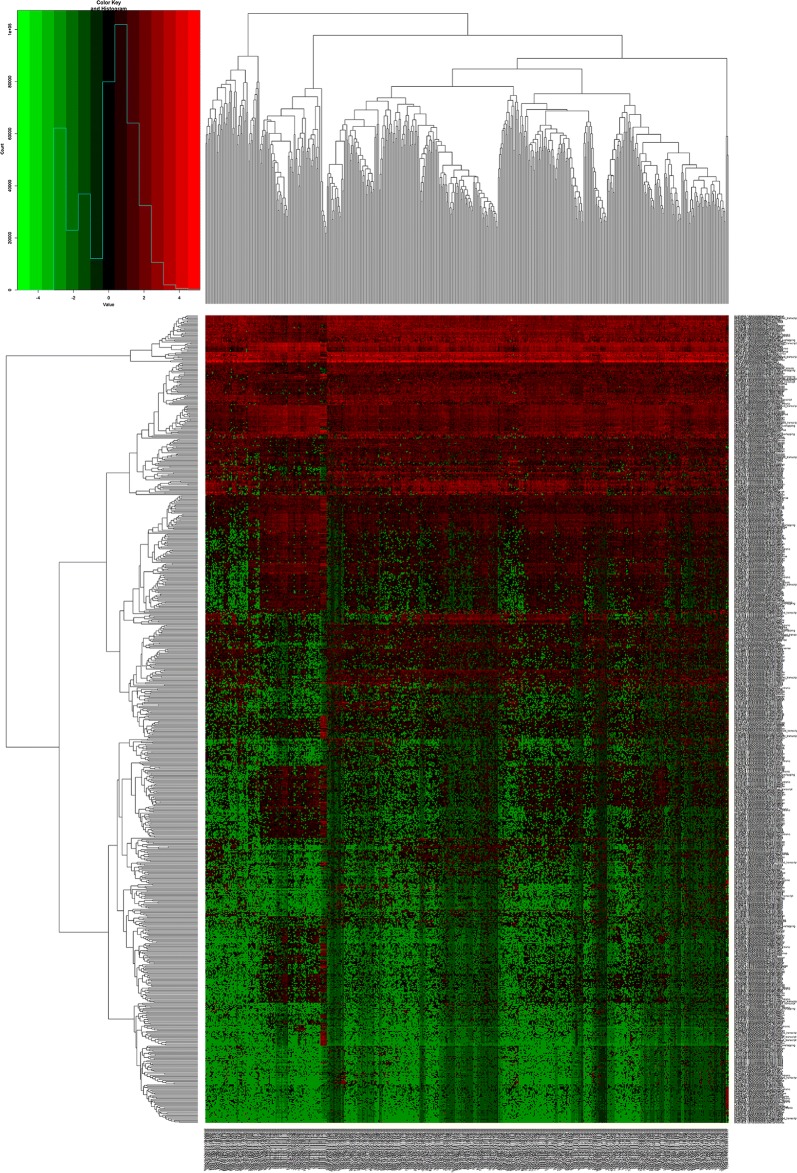
Heat maps of differential long non-coding RNAs (lncRNAs) in prostate cancer

**Fig. 3:**
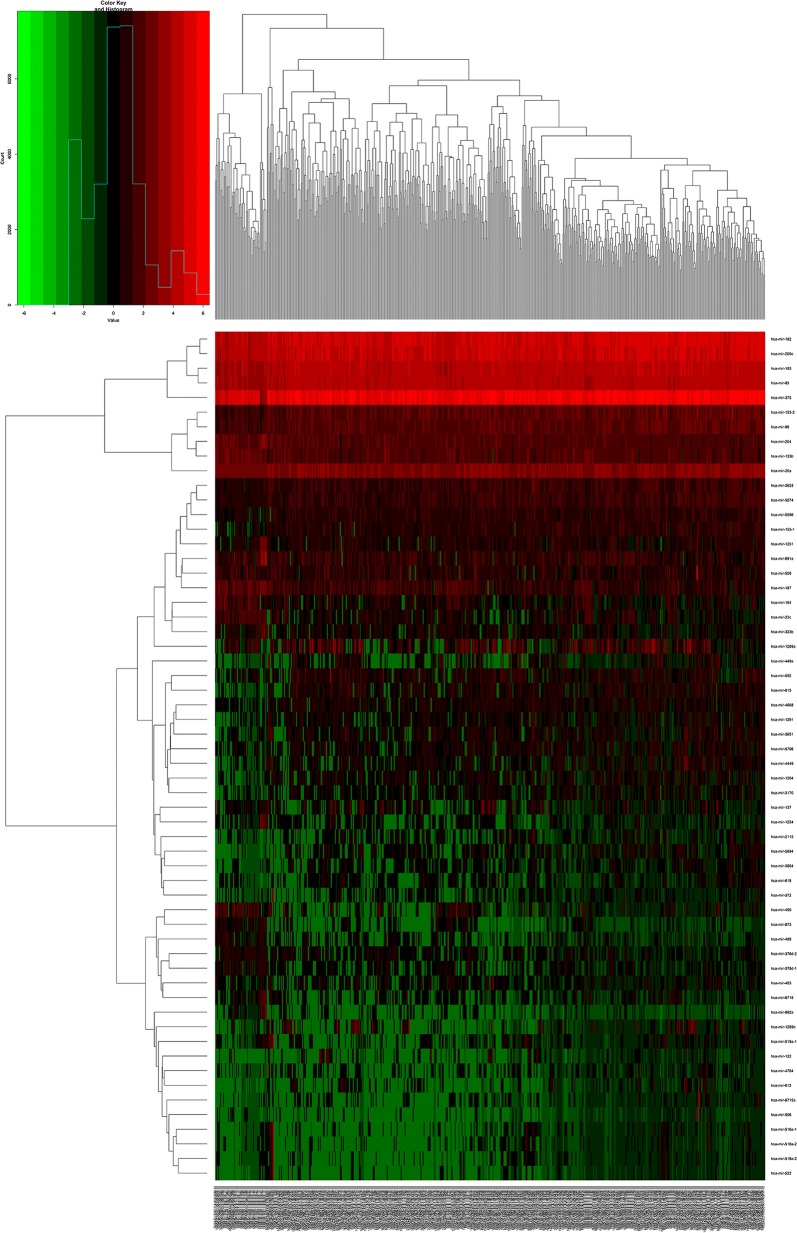
Heat maps of differential micro RNAs (miRNAs) in prostate cancer

A total of 19,660 mRNAs and 1881 miRNAs were identified from the TCGA database. Using fold-change > 1.5 and P value < 0.05 as cutoffs, we identified 1417 differentially expressed mRNAs (744 downregulated and 673 upregulated) (Fig. 2; Table 2) and 58 differentially expressed miRNAs (16 downregulated and 42 upregulated) (Fig. 3; Table 3).

**Table 2:** Top differential lncRNAs for prostate cancer

lncRNAs	logFC	logCPM	P value	FDR
EMX2OS	− 5.98142	9.023393	3.19E−214	2.45E−210
LINC02137	− 5.28623	4.228184	3.37E−164	1.30E−160
LINC01116	− 3.59594	6.425597	3.66E−153	9.36E−150
LINC00839	− 4.30616	5.907386	1.92E−152	3.69E−149
AL161645.1	− 4.9543	4.277951	3.17E−152	4.87E−149
AC012123.1	− 4.63826	4.053666	7.30E−150	9.35E−147
AL354793.1	− 5.45876	3.374845	3.31E−139	3.63E−136
LINC02385	− 5.3465	3.172287	5.10E−119	4.90E−116
HOXB-AS3	− 5.22943	5.154366	1.02E−109	8.72E−107
AC005674.1	− 3.98991	3.53896	1.86E−99	1.43E−96

**Table 3:** Top differential miRNAs for prostate cancer

miRNAs	logFC	logCPM	P value	FDR
hsa-mir-891a	− 4.85431879	4.002176579	1.55E−175	7.68E−173
hsa-mir-892a	− 5.149514076	− 0.257215178	3.34E−87	8.31E−85
hsa-mir-1224	− 3.661141606	0.125808001	1.07E−55	1.78E−53
hsa-mir-93	1.797800506	11.66361471	2.95E−55	3.67E−53
hsa-mir-23c	− 2.963769032	1.270390008	8.13E−53	7.72E−51
hsa-mir-1251	− 2.763816112	1.733525651	9.33E−53	7.72E−51
hsa-mir-204	− 1.84293445	4.58407729	1.85E−50	1.31E−48
hsa-mir-323b	− 2.318579119	0.539567334	8.77E−42	4.36E−40
hsa-mir-200c	1.584566628	13.50446843	2.04E−40	9.21E−39
hsa-mir-96	1.987872725	4.714552069	2.35E−39	9.75E−38

### Predictions of mRNAs and lncRNAs targeted by miRNAs

Next, we predicted the mRNAs and lncRNAs that were targeted by miRNAs, focusing on the relationship between the 58 differentially expressed miRNAs and 773 differentially expressed lncRNAs above. Only 13 of 58 differentially expressed miRNAs were predicted to target 63 of 773 differentially expressed lncRNAs.

**Fig. 4:**
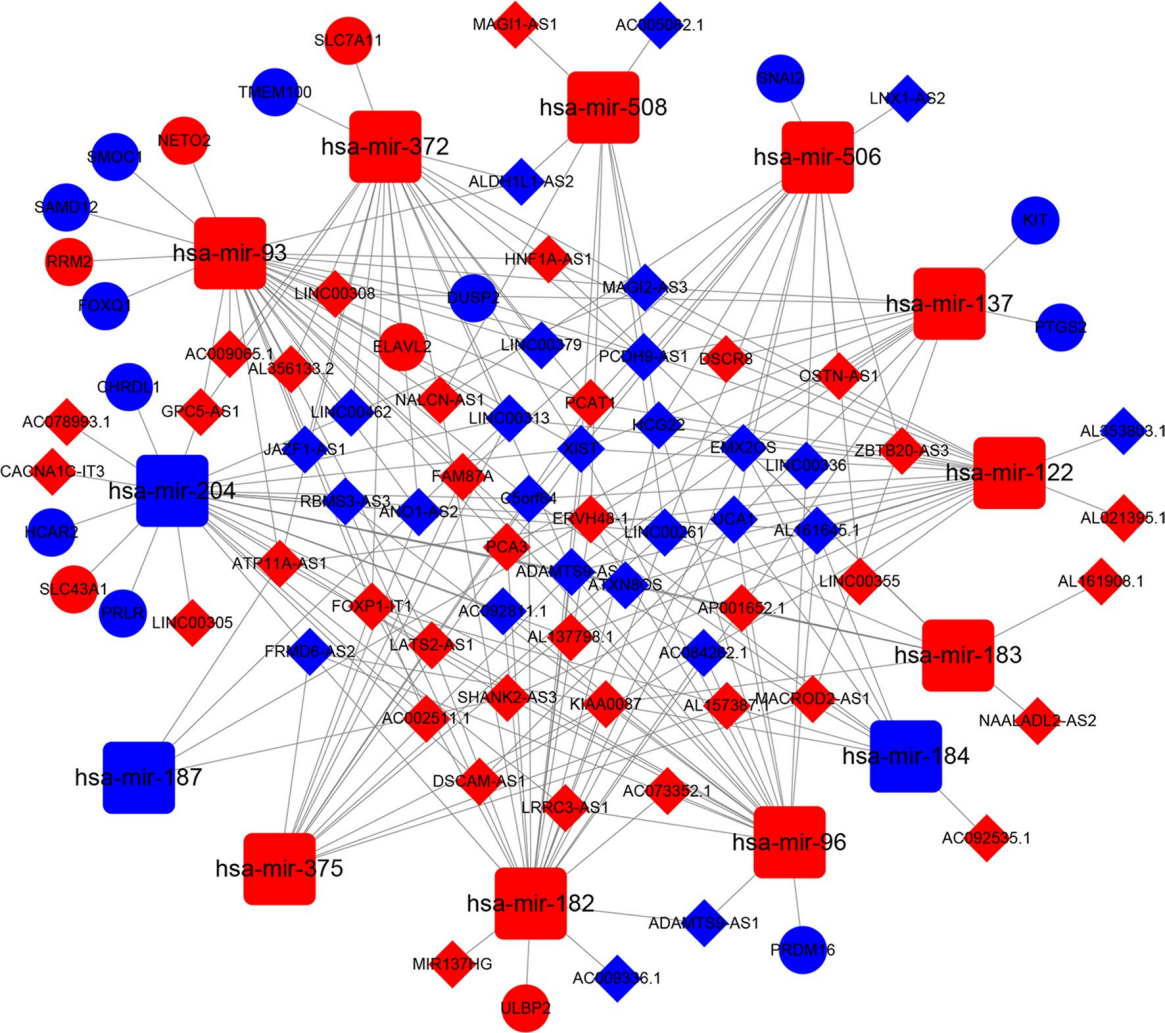
CeRNA network in prostate cancer. The blue nodes represent decreased expression, and the red nodes represent increased expression. Rectangles represent miRNAs, ellipses represent protein-coding genes, and diamonds represent lncRNAs; gray edges indicate lncRNA-miRNA-mRNA interactions

The relationships between these 13 differentially expressed lncRNA-targeting miRNAs were used to predict the targeted mRNAs using Targetscan, miRTarBase, and miRDB. Then, 13 prostate cancer-specific miRNAs were predicted to target the 644 mRNAs. After 644 mRNAs were found, the intersection of 644 mRNAs and 19,660 differentially expressed mRNAs between prostate cancer and adjacent normal prostate tissue were performed. Finally, 18 mRNAs were obtained from the 644 mRNAs. Overall, 63 lncRNAs, 13 miRNAs, and 18 mRNAs were selected to construct the lncRNA-miRNA-mRNA ceRNA network using Cytoscape 3.8.5 (Fig. 4; Tables 4 and 5).

**Table 4:** Representative interactions between lncRNAs and miRNAs for prostate cancer

lncRNA	miRNA
KIAA0087	hsa-mir-96, hsa-mir-182, hsa-mir-183, hsa-mir-204, hsa-mir-375
SHANK2-AS3	hsa-mir-96, hsa-mir-187, hsa-mir-204, hsa-mir-122
FAM87A	hsa-mir-96, hsa-mir-93, hsa-mir-506, hsa-mir-375
LINC00313	hsa-mir-93, hsa-mir-372, hsa-mir-187, hsa-mir-204, hsa-mir-122, hsa-mir-375
AC092811.1	hsa-mir-96, hsa-mir-182, hsa-mir-204, hsa-mir-93, hsa-mir-204
UCA1	hsa-mir-96, hsa-mir-182, hsa-mir-184, hsa-mir-122, hsa-mir-506
AP001652.1	hsa-mir-96, hsa-mir-137, hsa-mir-182, hsa-mir-183, hsa-mir-204
ATP11A-AS1	hsa-mir-93, hsa-mir-372, hsa-mir-96, hsa-mir-187, hsa-mir-122
NALCN-AS1	hsa-mir-93, hsa-mir-372, hsa-mir-182, hsa-mir-508, hsa-mir-506
ERVH48-1	hsa-mir-96, hsa-mir-137, hsa-mir-182, hsa-mir-184, hsa-mir-187, hsa-mir-508
MAGI2-AS3	hsa-mir-93, hsa-mir-372, hsa-mir-137, hsa-mir-204, hsa-mir-508, hsa-mir-122
PCAT1	hsa-mir-93, hsa-mir-372, hsa-mir-182, hsa-mir-122, hsa-mir-506, hsa-mir-375
FRMD6-AS2	hsa-mir-96, hsa-mir-182, hsa-mir-184, hsa-mir-204, hsa-mir-375
LINC00261	hsa-mir-182, hsa-mir-183, hsa-mir-204, hsa-mir-508, hsa-mir-506, hsa-mir-375

**Table 5:** Representative interactions between miRNAs and mRNAs for prostate cancer

miRNA	mRNA
hsa-mir-122-5p	HECW2, DUSP2, ORC2, CLIC4, SLC7A1, BROX, SLC52A2, PKM, NFX1, ANKRD13C, PRKRA, GNPDA2, GYS1, CCNG1, PIP4K2A, RBL1, RBM43, CCDC43, TNRC6A, ALDOA, FAM117B, G6PC3, NPEPPS, TGFBRAP1, HECTD3, SLC9A1, AKT3, PHF14, GALNT3, NT5C3A, P4HA1, FUNDC2
hsa-mir-137	CTBP1, MITF, HNRNPDL, SLC1A5, EOGT, PTGS2, NCOA3, GLO1, YTHDF3, GLIPR1, FMNL2, RREB1, SNRK, E2F6, KIT, DR1, YBX1, GIGYF1, SFT2D3, RORA, AGO4, NCOA2, CSE1L, LIMCH1, PXN, PAPD7, KDM1A, ESRRA, ZNF326
hsa-mir-182-5p	FLOT1, SESN2, BDNF, PLEKHA8, MTSS1, CITED2, CLOCK, MITF, NR3C1, TCEAL7, FBXW7, THBS1, EVI5, FGF9, FOXO3, KDM5A, CHL1, NPTX1, ADCY6, ULBP2, HOXA9, LSM14A, NUFIP2, PRKAA2, RARG, BRWD1, CYLD, TP53INP1, FOXF2, RECK
hsa-mir-183-5p	GLUL, ARHGAP21, FOXN2, LRP6, SRSF2, KIF2A, RCN2, TMED7, NR3C1, FOXO1, SH3D19, PPP2CB, KLHL24, EZR, RALGDS, SUCO, AKAP12, FAM217B, ZEB1, CTDSPL, KLRD1, ARFGAP2, KIF5C, CCNB1, NUFIP2, DAP, ITGB1, KLHL23, PDCD4, FAM175B, CELF1, IDH2, GNG5, PRRC1, PDCD6
hsa-mir-184	LRRC8A
hsa-mir-187-3p	DYRK2
hsa-mir-204-5p	ZFHX3, CREB5, CCNT2, RAB22A, CAPRIN1, M6PR, USP47, TGFBR2, ARAP2, AKAP1, MAPRE2, HAS2, HNRNPA2B1, JARID2, KLHL40, ANGPTL2, PHF13, SH3PXD2A, SAMD5, AP1S2, HOXC8, MAP1LC3B, SP1, RAB40B, RUNX2, FOXC1, COL5A3, MBNL1, SIRT1, CHRDL1, PPP3R1, IKZF2, FARP1, SGPL1, ARHGAP29, PRLR, ZCCHC24, PRDM2, AP1S1, TPPP, ANKFY1, CDH2, ITPR1, SERINC3, SLC43A1, RAB10, WWC3, ANKRD13A, EDEM1, ZBTB22, NPTX1, SLC22A6, ALPL, SYNJ2BP, TMTC2, NTRK2, BCL2, PTPRT, THRB, ELOVL6, SPOP, TCF12, EZR, CHORDC1, HCAR2, IL11, SLC39A9, BIRC2
hsa-mir-372-3p	ZNF532, WEE1, LATS2, SLC22A23, DUSP2, RAB11FIP1, TMEM100, FAM102B, SLAIN2, NR2C2, FEM1C, KLF3, MED17, DPP8, HABP4, MBNL2, ARID4B, PLA2G12A, ATAD2, PFKP, ULK1, CLIP4, TGFBR2, MKNK2, CUL3, ZNF385A, UNK, SERF1B, YOD1, TFAP4, SAR1B, PSD3, CADM2, DAZAP2, ZFYVE26, SIK1, IGF1R, TAOK1, IRF2, MIXL1, SBNO1, SUZ12, TXNIP, SUCO, ELAVL2, INO80D, GALNT3, LEFTY1, BTG1, MPP5, TMEM19, ELK4, HIP1, CREBRF, REST, TIMM17A, FOXJ2, OSTM1, MINK1, RHOC, RAB22A, IRAK4, LIMA1, HMBOX1, SH3GLB1, GNB5, SLC7A11, CCSAP, TNKS2, TRPS1, PAK2, KREMEN1, PTPDC1, NFIB, SERF1A, FBXL7, CPT1A, TNFAIP1, KPNA2
hsa-mir-375	ELAVL4, RLF
hsa-mir-506-3p	CD151, PI4K2B, NUFIP2, TMEM41A, SLC16A1, PARP16, PRR14L, CHSY1, SFT2D3, PTBP3, LRRC1, NEK9, GXYLT1, SNX18, AMOTL1, VIM, MYO10, SCAMP4, PTBP1, ZWINT, CREBRF, LRRC58, SNAI2
hsa-mir-93-5p	MKNK2, KLF3, CDKN1A, GID4, SCAMP2, MAP3K2, BRMS1L, EPS15L1, SAMD12, ZNF800, PANK3, HEG1, CEP97, PPP3R1, TMEM167A, ZNF280B, ORMDL3, ZBTB18, CAPRIN2, RB1, PAFAH1B1, FBXO21, DNAJC27, FCHO2, CCDC71L, PRRG1, KLHL20, PARD6B, HAUS8, MASTL, FNBP1L, NIPA1, NRIP3, CENPQ, BMP8B, SERF1B, POLQ, RCCD1, NETO2, JAK1, NR2C2, RBBP7, PURA, MTF1, DDHD1, NKIRAS1, TET3, FRS2, MED12L, PTPN4, ADARB1, NAGK, SMAD5, AGO1, PTGFRN, HSPA8, FBXO48, PIP4K2A, TMEM64, FJX1, SOWAHC, ANKH, RRAGD, PGP, CAMTA1, DUSP2, ZBTB9, FAM57A, ZADH2, KLHL28, C9orf40, ARHGAP12, SQSTM1, RABEP1, REST, RUNX3, ARHGAP1, SLAIN2, SGTB, BTBD7, SERF1A, F3, STK17B, SFXN5, RAP2C, ZBTB41, ITCH, SEMA4B, KATNAL1, UBE2Q2, RAB10, SALL3, TMEM242, CYBRD1, RAB11FIP1, LYSMD3, TRIP10, GINS4, FAM210A, SEMA7A, STX6, KAT2B, DAB2, STAT3, ENPP5, KLF10, PPP6C, PFKP, OSTM1, RBM12B, IKZF4, DENND5B, FAM102A, CEP170, KIAA0513, TBC1D20, CEP57, CNOT6L, SACS, ZBTB4, ABHD2, POLR3G, ZFP91, FBXO31, KPNA2, FIGNL1, C3orf38, E2F5, TMEM168, RAB22A, KIAA1191, ITGB8, CRK, ZNFX1, CNOT4, GBF1, PLXNA1, TNFAIP1, MAPRE3, SHOC2, HIP1, PIP4K2C, ASF1A, LASP1, EZH1, NABP1, ANKRD33B, HBP1, BMPR2, ZNF107, USP3, RRM2, MFN2, TFAM, HMGB3, LIMA1, RHOC, EPHA4, PLEKHO2, SMOC1, RPS6KA5, ZFYVE9, UXS1, EIF5A2, OXR1, UNKL, KMT2B, FYCO1, MAP3K3, PRR14L, FOXJ2, CNOT7, TANC1, PGM2L1, VPS26A, MCL1, RAPGEF4, KIAA0922, GNB5, VPS13C, EGR2, GPATCH2, ARHGAP35, FAXC, KLF9, EPHA7, SYBU, REEP3, ATL3, CLOCK, ANKRD13C, CAPN15, SOX4, SKIL, NPAT, ATAD2, U2SURP, SESN3, RPF2, FAM126B, FAM46C, KIF23, AKTIP, MIDN, TMEM123, ATG16L1, TOPORS, EGLN3, RAB5B, ABCA1, FOXQ1, NRBP1, TGFBR2, TNKS1BP1, PITPNA, GOLGA1, MORF4L1, SCAMP5, SERTAD2, HAS2, SPOPL, ELK4, RGMB, TMEM127, RNF145, NIN, TNKS2, SLC2A4, CHAF1A, CASP2, TMEM138, WDR37, FAM117B, USP32, CERCAM, WAC, TOLLIP, CFL2, SPRED1, ARAP2, DNM1L, TXLNA, RPA2, MTMR3, SGMS1, TWF1, TP53INP1, C7orf43, CDC37L1, TXNIP, E2F1, GPR137C, TRIM37, YOD1, CSNK1G1, PPP6R3, GNS, FRMD6, PHF6, ZNF202, PLS1, BICD2, CCSER2, CMPK1, SRSF2, CIT, CRY2, SNX16, HIF1A, EIF4H, RUNDC1, C14orf28, LPGAT1, CCND1, 2-Sep, PXK, RORA, NDEL1, VLDLR, LYST, TNFRSF21, UNK, ANKIB1, CREB1, STK11, ATG14, SLC16A9, MLXIP, SIKE1, FOXJ3, GOLGA2, PPP1R3B, ZFYVE26, MYO19, IRF1, BTG3, KIAA1147, BNIP2, FEM1C, PKD2, ZNF217, MINK1, PHTF2, GIGYF1, ZNF148, ANKRD50, IRAK4, ARID4B, SLK, ERAP1, NFAT5, ANKRD12, ULK1, ZC3H12C, PPP1R15B, FBXL5, PAPOLA, TMEM245, CCNG2, DNAJB9, RLIM, DPYSL2, TADA2B, ANKRD52, PTPDC1, KLF11, PDZD11, SASH1, CHIC1, ANKRD29, IFNAR1, EFCAB14, CHD9, OCRL, OSR1, NUP35, ACSL4, RUFY2, ZNF532, MAPK1, SSX2IP, HMBOX1, DDX5, UBXN2A, PKNOX1, NCOA3, LDLR, SNTB2, GAB1, USP28, UBE2J1, DUSP8, MCC, BTBD10, FAM129A, E2F2, ELAVL2, PDE3B, SLC29A2, GPAM, MAPK9, TUSC2, SH3PXD2A, SSH2, NACC2, APBB2, ZBTB7A, CLIP4, TMBIM6, NHLRC3, MFSD8, PTP4A1, SIK1, TSG101, PBX3, SUCO, DYNC1LI2, BBX, PHC3, LAPTM4A, NPAS2, STYX, EEA1, SLC22A23, NAA30
hsa-mir-96-5p	JAZF1, SLC25A25, KRAS, CNNM3, MAP3K3, EDEM1, SLC1A1, SNX7, STK17B, FOXO1, TMEM170B, APPL1, PRKAR1A, MBD4, PRDM16, ADCY6, ZEB1, EIF4EBP2, SCARB1, REV1, TSKU, ABCD1, SNX16, PPP1R9B, TRIB3, NHLRC3, PRKCE, DDIT3, MED1, CASP2, SIN3B, CCNG1, FRS2, PROK2, DDAH1, ALK, ASH1L, MORF4L1, SLC39A1

### Survival analysis with differentially expressed lncRNAs

**Fig. 5:**
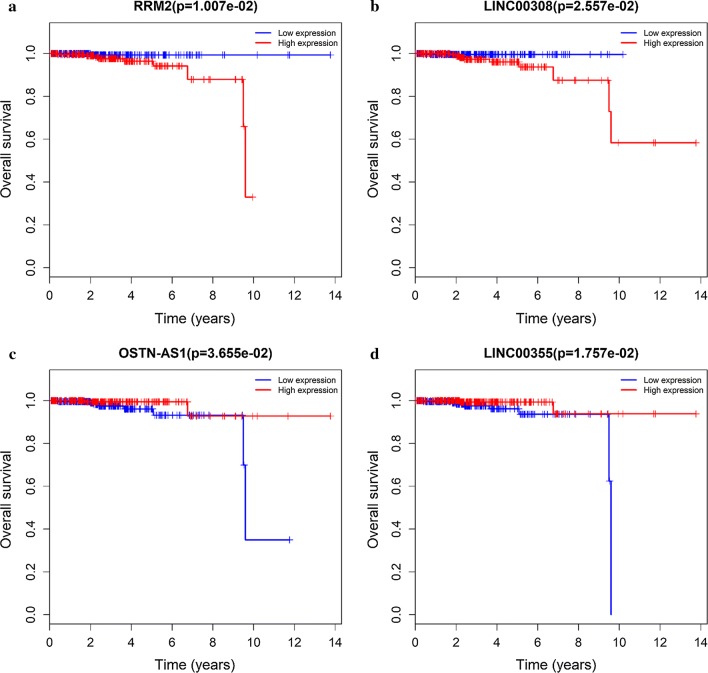
Kaplan–Meier survival curves for 1 protein-coding gene RRM2 (**a**) and 3 lncRNAs LINC00308 (**b**), OSTN-AS1 (**c**) and LINC00355 (**d**) associated with overall survival in prostate cancer. P < 0.05

To examine the relationship between the differentially expressed lncRNAs and the prognosis of patients with prostate cancer, the link between overall survival and the 63 differentially expressed lncRNAs in prostate cancer patients was analyzed by Kaplan–Meier method. Three of 63 differentially expressed lncRNAs were linked to the prognosis in prostate cancer: LINC00355 and lncRNA OSTN-AS1 were positively associated with overall survival, whereas LINC00308 correlated negatively with it (log-rank P < 0.05) (Fig. 5).

### Establishment of a 4-mRNA signature associated with overall survival in prostate cancer patients

Univariate Cox regression analysis was first used to identify prognosis-related mRNAs, identifying 21 mRNAs that were significantly related to overall survival (P < 0.01). Then, multivariate Cox regression was performed, and 4 mRNAs were ultimately selected to establish a predictive model. The predictive model was defined as the linear combination of the expression levels of the 4 mRNAs, which were weighted using the corresponding relative coefficient in the multivariate Cox regression as follows: survival risk score = (0.420 × expression value of HOXB5 + 0.794 × expression value of GPC2 + 0.947 × PGA5 + 0.473 × AMBN). All 4 mRNAs had positive coefficients in the Cox regression analysis, indicating that their high expression was associated with shorter overall survival in prostate cancer patients.

### Risk stratification and ROC curve analysis

**Fig. 6:**
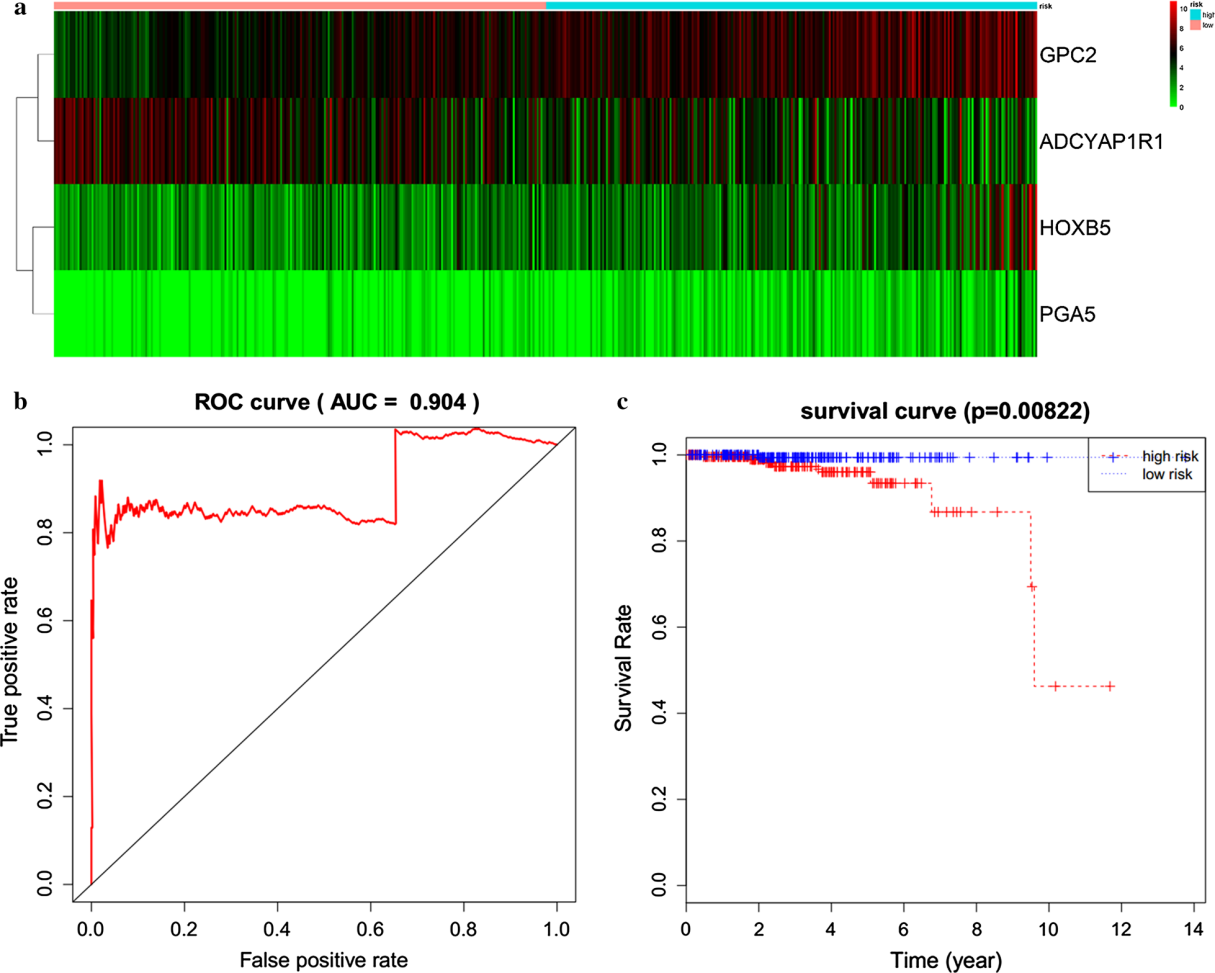
Prognostic evaluation of the 4-mRNA signature in prostate cancer patients. **a** The distribution of mRNA-related survival risk scores and heatmap of the 4 prognostic mRNAs. **b** Kaplan–Meier analysis of overall survival in prostate cancer patients with the 4-mRNA signature. **c** ROC curve analysis of the 4-mRNA signature

The 4-mRNA expression-based survival risk score was used to assign patients into a low-risk or high-risk group using a median risk score of 0.9558 as the cutoff. Ultimately, a total of 247 patients were assigned to the high-risk group, versus 248 in the low-risk group (Fig. 6a). The Kaplan–Meier curves for overall survival demonstrated that there was a significant difference between the 2 groups, based on the 4 mRNAs (Fig. 6b). The 5-year and 10-year overall survival rates were 96.0% and 46.3% in the high-risk group, respectively. The prognostic power of the 4-mRNA signature was evaluated using the area under the ROC curve. In this study, the area under the ROC curve was 0.904, indicating good sensitivity and specificity of the 4-mRNA signature in predicting survival in prostate cancer patients (Fig. 6c; Table 6).

**Table 6:** Multivariate Cox regression analysis of 4 prognostic mRNAs associated with overall survival in prostate cancer patients

mRNA	coef	exp(coef)	se(coef)	z	P
HOXB5	0.42	1.522	0.155	2.7	0.00688
GPC2	0.794	2.213	0.382	2.08	0.03735
ADCYAP1R1	− 0.396	0.673	0.249	− 1.59	0.11195
PGA5	0.947	2.577	0.286	3.31	0.00094
AMBN	0.473	1.605	0.187	2.53	0.01139

### Functional assessment

**Fig. 7:**
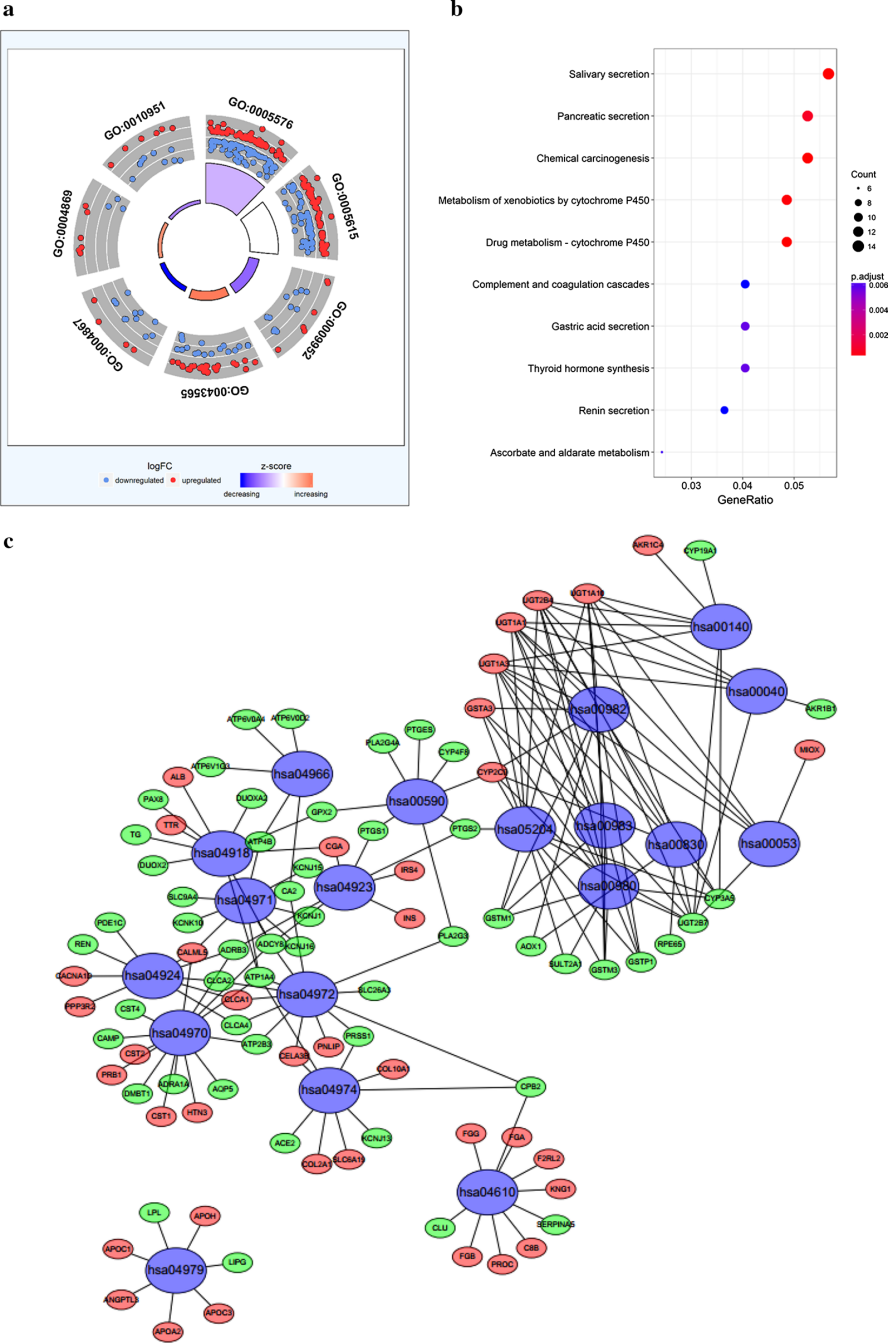
Plot of enriched GO and KEGG terms for the differentially expressed genes. **a** Plot of enriched GO terms for differentially expressed mRNAs. **b**, **c** Plot of enriched KEGG pathways for differentially expressed mRNAs. *GO* gene ontology, *KEGG* kyoto encyclopedia of genes and genomes, *FDR* false discovery rate

The functions of the differentially expressed mRNAs in the ceRNA network were determined using DAVID bioinformatics resources. The results demonstrated that 7 GO terms and 19 enriched KEGG pathways were involved in the ceRNA network (Fig. 7; Table 7).

**Table 7:** KEEG pathways enriched by mRNAs

Pathway ID	Description	P-value	Count
hsa04970	Salivary secretion	1.38E−06	14
hsa05204	Chemical carcinogenesis	2.65E−06	13
hsa00982	Drug metabolism—cytochrome P450	2.77E−06	12
hsa00980	Metabolism of xenobiotics by cytochrome P450	5.08E−06	12
hsa04972	Pancreatic secretion	1.58E−05	13
hsa04918	Thyroid hormone synthesis	0.000156	10
hsa04971	Gastric acid secretion	0.000175	10
hsa00053	Ascorbate and aldarate metabolism	0.000213	6
hsa04610	Complement and coagulation cascades	0.00027	10
hsa04924	Renin secretion	0.000276	9
hsa00830	Retinol metabolism	0.000311	9
hsa00140	Steroid hormone biosynthesis	0.000696	8
hsa00040	Pentose and glucuronate interconversions	0.000795	6
hsa00590	Arachidonic acid metabolism	0.000974	8
hsa00983	Drug metabolism—other enzymes	0.001185	9
hsa04979	Cholesterol metabolism	0.001238	7
hsa04966	Collecting duct acid secretion	0.001754	5
hsa04923	Regulation of lipolysis in adipocytes	0.00196	7
hsa04974	Protein digestion and absorption	0.002961	9

## Discussion

Differentially expressed lncRNAs that correlated significantly with OS were identified by constructing an lncRNA-miRNA-mRNA ceRNA network, based on specific criteria in a large sample of prostate cancer patients in the TCGA database. Thus, there are potential interactions between mRNAs, lncRNAs, and miRNAs in the progression and metastasis of prostate cancer. In this study, ceRNA networks for prostate cancer were built by bioinformatics prediction and correlation analysis of data on significantly differentially expressed mRNAs, lncRNAs, and miRNAs. Further, considering the associations between cancer-specific ceRNAs and clinical characteristics, 3 lncRNAs (LINC00308, OSTN-AS1 and LINC00355) were related to the clinical prognosis. Moreover, 4 mRNAs (HOXB5, GPC2, PGA5, and AMBN) which screened to establish a predictive model were also associated with the clinical prognosis. Both 3 lncRNAs and 4 mRNAs are important because these RNAs are associated with overall survival of patients. These RNAs might provide more powerful prognostic information in predicting survival in prostate cancer.

The mechanisms that underlie the progression and metastasis of prostate cancer remain unknown. However, our understanding of the genesis and characteristics of prostate cancer has grown because of the development of high-throughput sequencing and bioinformatics. Recently, Liu et al. [17] revealed that miRNA genes can be considered tumor suppressor genes and novel oncogenes that are involved in the progression and metastasis of carcinomas. Liu et al. [17] also demonstrated that miR-141 employs several mechanisms to reduce the growth and metastasis of prostate cancer. Liu et al. [18] reported that the microRNA miR-34a inhibits the regeneration and metastasis of prostate cancer by repressing CD44 directly. Tinay et al. [19] demonstrated that 3 miRNAs are significantly overexpressed in serum from prostate cancer patients versus those without cancer. In this study, 58 miRNAs were significantly differentially expressed in prostate cancer compared with adjacent non-tumorous tissues.

lncRNAs are potential biomarkers in carcinogenesis and have significant advantages as diagnostic and prognostic biomarkers [20]. Previous research has confirmed that differentially expressed lncRNAs correlate with the progression and metastasis of carcinomas [21, 22]. Ramnarine et al. [23] reported that the lncRNAs FENDRR, H19, LINC00514, LINC00617, and SSTR5-AS1 are involved in the development of neuroendocrine prostate cancer. Zhang et al. [24] found that cell proliferation in hormone-refractory prostate cancer is promoted by the lncRNA PCGEM1. In this study, 773 lncRNAs were identified. LINC00355 and OSTN-AS1 were positively associated with overall survival, whereas LINC00308 correlated negatively with overall survival. LINC00355, OSTN-AS1, and LINC00308 were included in the ceRNA network, suggesting that these lncRNAs play an important role in the progression and prognosis of prostate cancer.

Only 1 of 18 differentially expressed mRNAs (RRM2), which constructed of ceRNA networks, were significantly associated with overall survival in prostate cancer. Although RRM2 has been studied in colorectal cancer [25], non-small cell lung cancer [26], pancreatic cancer [27], adrenocortical cancer [28], and cervical cancer [29]. However, the role of RRM2 in prostate cancer has not been established yet. In this study, the higher expression of RRM2 was associated with worse survival outcome in prostate cancer. Chang et al. [25] demonstrated that overexpression of RRM2 was associated with survival and recurrence in colorectal cancer patients with k-ras mutation. Yoshida et al. [30] found that the upregulation of RRM2 was essential for the proliferation of colorectal cancer cell lines. Rahman et al. [26] indicated that knockdown of RRM2 was associated with apoptosis of head and neck squamous cell carcinoma and non-small cell lung cancer. These finds mentioned above suggested that RRM2 may be a potential prognostic targets in prostate cancer.

However, there are no reports on the correlation between LINC00308 and disease. Moreover, the function of LINC00308 has not been examined. Thus, the genes that are related to LINC00308 were predicted by constructing an lncRNA-miRNA-mRNA network. The results demonstrated that 2 miRNAs (has-mir-137 and has-mir-93-5p) are associated with LINC00308. The target genes of these 2 miRNAs were then predicted, resulting in 29 has-mir-137 target genes and 385 has-mir-93-5p target genes. We found three common hits between the target genes of these 2 miRNAs: RORA, GIGYF1, and NCOA3. Mocellin et al. [31] reported that RORA is significantly associated with the risk of breast carcinoma, prostate carcinoma, and lung carcinoma. Zhu et al. [32] also found that RORA is a common fragile site gene that is inactivated in several carcinomas and is involved in responses to cellular stress. Moretti et al. [33] reported that RORA is a molecular target for the development of chemotherapeutic strategies for prostate carcinoma. Ajiro et al. [34] demonstrated that the phosphorylation of Akt at Ser 473 is significantly reduced after GIGYF1 knockdown in breast cancer cell lines. Tong et al. [35] revealed that NCOA3 is overexpressed in human hepatocellular carcinoma specimens and promotes the proliferation of human hepatocellular carcinoma. Ngollo et al. [36] showed that NCOA3 is upregulated in prostate cancer compared with normal prostate tissues. Moreover, the expression of NCOA3 also correlates with Gleason score, clinical stage, and PSA levels.

Conventional prognostic systems generally make insufficient predictions for risk stratification and estimations of clinical outcome because of the heterogeneity between patients. Thus, in recent decades, much effort has been made to establish a novel prognostic model to improve the prediction of survival in prostate cancer patients [37–39]. In this study, we generated a 4-mRNA signature that predicted the clinical outcome of prostate cancer. To the best of our knowledge, this is the first mRNA-related predictive model that is based on TCGA RNA-seq data from 495 prostate cancer patients. These 4 mRNAs were identified to establish a predictive model that is based on their linear combination. A significant difference of survival rate was observed between the high-risk and low-risk groups. In the ROC analysis, the AUC was 0.904, indicating high sensitivity and specificity of the mRNA signature. The GCP2 has been explored in several studies [40–42]. However, the role of GCP2 in prostate cancer has not been elucidated yet. Dráberová et al. [41] reported that the immunoreactivity of GCP2 was significantly increased in glioblastoma cells than that in normal brains cells. The GCP2 was also related to the progress of the microvascular proliferation. The dysregulation of GCP2 in glioblastomas may also associated with the alteration of transcriptional checkpoint activity.

The GO term analysis demonstrated that the differentially expressed mRNAs were involved primarily in sequence-specific DNA binding, negative regulation of endopeptidase activity, anterior/posterior pattern specification, extracellular space, extracellular region, cysteine-type endopeptidase inhibitor activity, and serine-type endopeptidase inhibitor activity. Furthermore, the enriched KEGG pathways of the differentially expressed mRNAs included salivary secretion, pancreatic secretion, chemical carcinogenesis, metabolism of xenobiotics by cytochrome P450, drug metabolism-cytochrome P450, complement and coagulation cascades, gastric acid secretion, thyroid hormone synthesis, renin secretion, and ascorbate and aldarate metabolism.

This study has some limitations. Although the data obtained from TCGA database represent an important tool for complex analyzes of biomarkers, it is known that they are produced by extremely heterogeneous samples. All data obtained and statistically analyzed in this study were not validated on representative samples subsequently in this study. Following are some reasons. On the one hand, the original design of this study was using varieties of bioinformatics tools and databases to dig useful and potential targeted mRNAs, miRNAs, and lncRNAs which associated with the prognostic outcomes. On the other hand, we aimed to explore mRNA signatures that predict survival in prostate cancer. To the best of our knowledge, some mRNAs are not transcriptable, which means some mRNAs-related proteins cannot be detected in the immunohistochemistry assay. Third, the prostate cancer tissue and health prostate tissue are difficult to distinguish in the fresh pathological specimens. Thus, it is very difficult for us to do further validation based on the fresh pathological specimens assay of prostate cancer.

## Conclusion

In conclusion, we identified three differentially expressed lncRNAs that potentially predict overall survival in prostate cancer patients by analyzing the lncRNA, mRNA, and miRNA profiles in the TCGA database using a ceRNA network. The underlying mechanisms of these lncRNAs in prostate cancer should be determined.

## Abbreviations

miRNA: microRNA
mRNA: messenger RNA
lncRNA: long non-coding RNA
CRPC: castration-resistant prostate cancer
ceRNAs: competing endogenous RNAs
GDC: the genomic data commons data portal
TCGA: the genomic data commons data portal and the cancer genome atlas database
DAVID: database for annotation, visualization, and integrated discovery
GO: gene ontology database
KEGG: the kyoto encyclopedia of genes and genomes
ROC curve: receiver operating characteristic curve
AUC: area under curve
PSA: prostate-specific antigen
